# The differential effects of green tea on dose-dependent doxorubicin toxicity

**DOI:** 10.3402/fnr.v59.29754

**Published:** 2015-12-21

**Authors:** Slawomir Mandziuk, Renata Gieroba, Agnieszka Korga, Wlodzimierz Matysiak, Barbara Jodlowska-Jedrych, Franciszek Burdan, Ewa Poleszak, Michał Kowalczyk, Luiza Grzycka-Kowalczyk, Elzbieta Korobowicz, Aleksandra Jozefczyk, Jaroslaw Dudka

**Affiliations:** 1 Department of Pneumology, Oncology and Alergology, Medical University of Lublin, Lublin, Poland; 2 Independent Medical Biology Unit, Medical University of Lublin, Lublin, Poland; 3 Department of Histology and Embryology, Medical University of Lublin, Lublin, Poland; 4 Department of Anatomy, Medical University of Lublin, Lublin, Poland; 5 Department of Applied Pharmacy, Medical University of Lublin, Lublin, Poland; 6 1st Department of Anaesthesiology and Intensive Care, Medical University of Lublin, Lublin, Poland; 7 1st Department of Radiology and Nuclear Medicine, Medical University of Lublin, Lublin, Poland; 8 Department of Clinical Pathomorphology, Medical University of Lublin, Lublin, Poland; 9 Department of Pharmacognosy with Medicinal Plant Unit, Medical University of Lublin, Lublin, Poland

**Keywords:** cardiotoxicity, doxorubicin, green tea, catechins, oxidative stress

## Abstract

**Background:**

Doxorubicin (DOX) is an anticancer drug displaying cardiac and hepatic adverse effects mostly dependent on oxidative stress. Green tea (GT) has been reported to play a protective role in diseases resulting from oxidative stress.

**Objective:**

The objective of this study was to evaluate if GT protects against DOX-induced oxidative stress, heart and liver morphological changes, and metabolic disorders.

**Methods:**

Male Wistar rats received intraperitoneal injection of DOX (1.0 or 2.0 mg/kg b.w.) for 7 weeks or concomitantly GT extract soluble in drinking water.

**Results:**

There were multidirectional effects of GT on blood metabolic parameters changed by DOX. Among all tested biochemical parameters, statistically significant protection of GT against DOX-induced changes was revealed in case of blood fatty acid–binding protein, brain natriuretic peptide, and superoxide dismutase.

**Conclusion:**

DOX caused oxidative stress in both organs. It was inhibited by GT in the heart but remained unchanged in the liver. DOX-induced general toxicity and histopathological changes in the heart and in the liver were mitigated by GT at a higher dose of DOX and augmented in rats treated with a lower dose of the drug.

Doxorubicin (DOX) is an effective anticancer drug that causes serious adverse effects (1–4). One of them is irreversible, delayed cardiomyopathy (5), appearing even years after completing the treatment, impossible to cure, and finally leading to death in a small percentage of patients taking the maximal advised dose (6–8). DOX-induced delayed cardiotoxicity is thought to be a complex multifactorial process, in which oxidative stress plays a pivotal role (9, 10). It might be postulated that reactive oxygen species (ROS), produced in DOX presence (11–13), codes death program in cardiomyocytes mitochondria, which results in a positive feedback loop between mitochondrial DNA (mtDNA) damage and mitochondrial ROS overproduction resulting in ‘snow-ball’ type augmentation of cardiac failure with the passage of long time since DOX excretion from the organism. For a long time, the symptoms of a developing heart dysfunction are clinically silent. However, with the progress of oxidative stress, cardiomyocytes mitochondria become insufficient, leading to heart failure (14–17). The important reason for making cardiomyocytes selectively susceptible to DOX toxicity is a relatively low level of enzymatic antioxidative defense – over a dozen to over 20% of liver activity (18, 19). Moreover, DOX reduces superoxide dismutase (SOD) activity and therefore decreases the cardiomyocytes antioxidative defense (20). Oxidative stress has been shown to cause depolarization of the mitochondrial membrane, resulting in apoptosis (21, 22). DOX-induced mitochondria oxidative stress is associated with cardiomyocyte programmed cell death (10, 11). Other consequences of ROS generation include necrosis, heart remodeling, and changes in the cells’ metabolism which are also observed in the presence of DOX (17, 23–27). Similarly, DOX-dependent ROS cellular effect could be expected in hepatocytes. Despite the relative high activity of the antioxidant system in the liver (18, 19), the liver activity of NADPH cytochrome P450 reductase, the key enzymes in DOX-dependent ROS generation, is extremely high (28, 29).

The main hypothesis of this work is that green tea (GT) protects the heart muscle and the liver from DOX-induced oxidative stress and adverse changes in metabolism. Because secondary morphological changes can be related to oxidative stress, it is also assumed that DOX-induced histological changes will be quenched by GT extract. GT is rich in polyphenols that are characterized by antioxidant properties (30–34). Among them, catechins attract the most attention because they display antioxidative activity in animals and humans (35, 36). These properties are related to free radical scavenging and iron complexing (37). The iron complexing prevents Fenton's reaction, which generates a more aggressive free radical (38, 39). Moreover, polyphenols-related changes in redox equilibrium affect cell metabolism. Polyphenols of GT modulate the activity of some enzymes responsible for cholesterol and triglycerides synthesis, for example, carboxylase acetyl-Co-A and reductase HMG-CoA – the main target for statins (40, 41).

The objective of this study was to test the ability of GT to protect against DOX-induced heart and liver oxidative stress, morphological changes, and metabolic disorders. The GT protection in DOX-mediated toxicity has attracted attention in recent years. Promising results were obtained in the studies on culture cardiomyocytes and rats in the acute toxicity model (17, 20 mg of DOX/kg b.w.) (42–44). In this study, a cumulative model with a smaller dose of the drug (1 and 2 mg/kg. b. w.) repeated seven times once a week was applied, since it is more relevant to the clinical conditions.

## Materials and methods

### Animals and treatment

The study was conducted as part of a large toxicological project (45–48), after being approved by the Local Bioethical Council of the Medical University in Lublin. The experiment was carried out on male rats of Wistar strain with an initial body mass of 165–195 g. The animals were given standard granulated feed LSM (Agropol, Poland) and were provided continuous access to water enriched with GT extract. All animals were kept in standardized controlled conditions.

All rats were randomly divided into five groups. After acclimatization, animals were treated intraperitoneally once a week, for 7 weeks, with DOX hydrochloride (Ebeve Arzneimittel Ges. m. b. H., Unterachl, Austria), diluted v/v 1:2 with sterile saline at a dose of 1 or 2 mg/kg b.w. The control group received sterile saline intraperitoneally. In the other groups, in addition to DOX, decaffeinated extract of GT form Life Extension (Fort Lauderdale, USA) was administered in drinking water. Dietary supplementation with GT was started 1 week prior to the administration of DOX and continued for the entire duration of the experiment. Water (250 mL) at a temperature below 100°C was added to the content of standardized GT extract capsules containing 710.5 mg polyphenols (including 326.25 mg of epigallocatechin gallate (EGCG)) and after 15 min, it was made up to a volume of 2 L. The estimated average daily dose of EGCG for one rat was 16.3 mg/kg b.w. The animals were randomly divided into control (*n*=8) and four study groups (*n*=6): I – saline (K); II – 1 mg of DOX/kg b.w. (1DOX); III – 1 mg of DOX/kg b.w. and GT extract (1DOX+GT); IV – 2 mg of DOX/kg b.w. (2DOX); V – 2 mg DOX/kg b.w. and GT extract (GT+2DOX).

The material for the study was collected from animals in shallow pentobarbital anesthesia, 96 h after the administration of the last dose of DOX. A blood sample for biochemical tests was aspirated from the left ventricle to the tubes with the activator of coagulation or containing the anticoagulant EDTA-K_3_. After about 0.5 h, samples were centrifuged for 4 min at 4,000 rpm at 4°C. Immediately after blood collection, samples for the morphological studies from liver (left lobe) and heart (left ventricle) were taken, fixed in 10% buffered formalin, and routinely processed to paraffin-fixed blocks for histological examinations. The sections of the heart and the liver for biochemical studies were washed with saline, dried, and frozen in liquid nitrogen. The samples were stored at −75°C. The obtained organ samples were thawed, washed in saline, dried, and then homogenized in 20 mM phosphate buffer at pH 7.4(m/v 1: 4). Preliminary homogenization of the cardiac muscle was carried out in a porcelain mortar. Then the material was homogenized using a homogenizer with a Teflon piston (8 min at 4,000 rpm). Sections of the liver were homogenized in identical conditions for 3 min without preliminary investigation. Homogenates from both organs were centrifuged for 20 min at 15,000×g at 4°C. The obtained supernatants were used for further studies.

### Evaluation of serum biochemical parameters

The analysis of serum biochemical markers included concentrations of total cholesterol, triglycerides, creatinine, uric acid, glucose, and urea, and activity of lactate dehydrogenase (LDH), creatine kinase (CK), alkaline phosphatase (ALP), aspartate aminotransferase (AST), and alanine aminotransferase (ALT) were determined using Liasys (Milan, Italy) biochemical analyzer, with commercial diagnostic Cormay kits (Lublin, Poland).

Fatty acid–binding protein (FABP) from Life Diagnostic (West Chester, USA), specific for the rat's heart and rat-specific brain natriuretic peptide (BNP) from Phoenix Pharmaceuticals (Burlingame, USA) concentrations were assessed in serum with ELISA commercial kits according to the manufacturer's instructions. The product of the catalytic reaction was spectrophotometrically detected at 450-nm using Power Wave XS of BioTek (Highland Park, USA).

### Evaluation of tissue markers for redox imbalance

Lipid peroxidation was determined in the supernatants of liver and cardiac homogenates using the commercial kit BIOXYTECH^®^ LPO-586 OxisResearch (Foster City, USA). The method was based on the measurement of malondialdehyde (MDA) and 4-hydroxyalkenals (4-HAE) concentration. For determination of MDA and 4-HAE, spectrophotometric method was used where the reaction of N-methyl-2-phenylindole with MDA and 4-HAE at 45°C produced a colored compound. Absorbance measurement was made using Power Wave XS at a wavelength of 586 nm.

The activity of SOD was determined using the oxidation reaction of 5, 6, 6a, 11b-tetrahydro-3, 9, 10-trihydroksybenzo[c]fluoran, which is mediated by SOD in an alkaline medium. The reaction product is a colored compound with the maximum absorbance at a wavelength equal to 525 nm. Interference with mercaptans, such as reduced glutathione, is prevented by pre-treatment of samples with 1-methyl-2 vinyl pyridine, which comes directly in the alkylation reaction with sulfhydryl compounds. The measurement of enzyme activity was performed by the kinetic method using Power Wave XS. The average value calculated for the control group was taken as 100%.

The concentration of oxidized and reduced form of glutathione (GSH and GSSG) was quantified using commercial kit GSH/GSSG- 412™ OxisResearch (Foster City, USA). This enzymatic method is based on the reaction of GSH with Ellman's reagent (5,5′-dithiobis-2-nitrobenzoic acid) which gave a color product with maximum absorbance at 412-nm. The concentrations of GSH, GSSG, total glutathione (GSH_t_), and GSH/GSSG ratio were assessed after measuring the speed of the reaction and establishing the calibration curves according to the manufacturer's instructions.

### Preparation of slides for histological evaluation

Histological slides (4-µm) obtained from paraffin blocks were routinely processed and stained with hematoxylin and eosin (H&E). Cardiac slides were additionally stained by means of Selye's method and liver slides were stained using van Gieson, paS (periodic acid-Schiff), and d-paS (diastase+paS) method.

### Statistical analysis

The obtained data were statistically analyzed using STATISTICA 8.0 software. The results are expressed as mean±standard deviation (M±SD). Continuous data were compared among the experimental groups using the Kolmogorov–Smirnov test. The statistical significance of differences between control and the other groups was evaluated either by Student's *t*-test or U Mann–Whitney test. To compare more than two groups, the one-way analysis of variance ANOVA and post hoc multiple comparisons on the basis of Tukey's HSD test were used. The value of *p*≤0.05 was considered as statistically significant.

## Results

There were no deaths or clinically visible drug-related behavioral and general condition changes (except body weight) among the studied rats. No gross organ abnormalities were seen during autopsy.

The mean body weight of rats treated with a lower dose of DOX was about half that of the control group, but this difference was not statistically significant (Table 1). A significant reduction of body weight was found among the animals exposed to a higher dose of the drug. The effect of GT in both of the above groups was divergent. In group 1DOX+GT, the adverse effect exacerbated, but in group 2DOX+GT, the DOX-induced the reduction of body weight was mitigated.

**Table 1:** The weekly relative differences of body mass [Δ%]

	The differences between subsequent weeks							
		
	1/2	2/3	3/4	4/5	5/6	6/7	7/8	M±SD of 1/2–7/8 weeks
Control	3.39	5.56	4.21	2.17	3.46	2.26	0.74	3.13±1.60
1DOX	3.35	1.62	0.39	−0.52	−0.23	3.58	2.05	1.46±1.65
1DOX+GT	4.20	−0.10	0.20	0.94	2.83	−1.54	−0.02	0.93±1.95*
2DOX	4.73	−1.87	−2.28	−2.38	−2.72	−4.89	−4.40	−1.97±3.17*
2DOX+GT	4.58	0.20	−1.63	−0.60	1.13	−3.83	−2.64	−0.40±2.76*

* *p*<0.05 vs control.

**Fig. 1:**
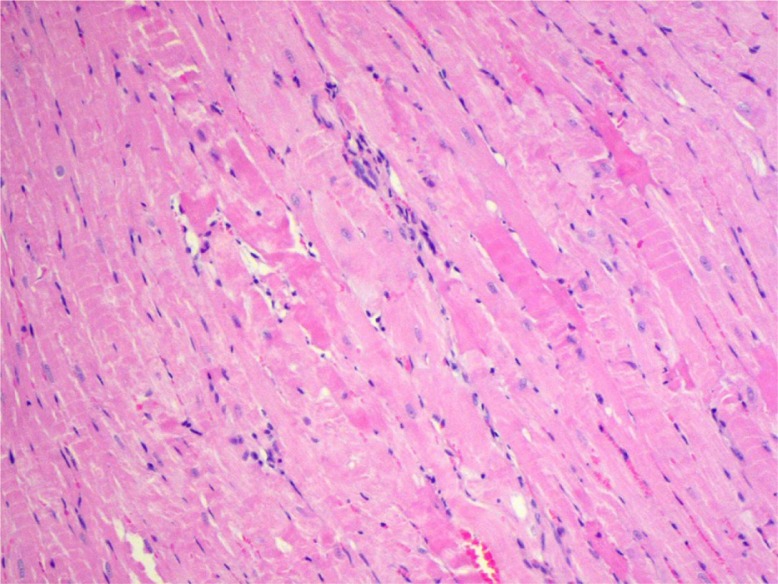
Interstitial edema and inflammatory infiltration between irregular, wavy-directed cardiomyocytes (H+E; objective mag.×20; group 2DOX).

Histologically, an irregular direction of cardiomyocytes and occasionally observed parenchymatous degeneration and interstitial edema were the only cardiac abnormalities revealed in the untreated control group (Table 2, Fig. 1). Their incidence was higher among the animals exposed to DOX. In this group, a high, dose-dependent occurrence of cardiomyocytes, eosinophilic and vacuolar degeneration, as well as myocardial necrosis usually with concomitant inflammatory infiltration were seen. Co-administration of GT increased the incidence of cardiac pathologies among the animals exposed to a low dose of DOX, and decreased in rats exposed to a higher dose of the drug.

**Table 2:** Histopathological cardiac changes in animals exposed to doxorubicin (DOX) with or without green tea (GT)

	n	Eosinophilic degeneration	Parenchymatous degeneration	Vacuolar degeneration	Irregular direction of cardiomyocytes	Pycnotic nuclei of cardiomyocytes	Interstitial edema	Necrosis	Inflammatory infiltration
Control	8	0	1	0	3	0	1	0/0^a^	0/0^b^
1DOX	6	2	3	2	3	1	3	0/4	0/4
1DOX+GT	6	5	3	2	4	0	1	1/2	1/1
2DOX	6	6	4	3	5	2	5	4/2	4/2
2DOX+GT	6	2	3	3	4	2	0	0/2	0/4

A single animal may be represented more than once in the listing of individual histological changes.

a Massive necrosis/changes limited to single cardiomyocytes.

b Massive inflammatory infiltration/disseminate mononuclear cells between cadiomyocytes.

Histological hepatic abnormalities were rarely proved in both untreated control and xenobiotic-exposed groups (Table 3). In untreated control, a single case of minute inflammatory infiltration around central vein was found. In groups exposed to DOX a high incidence of eosinophilic and parenchymatous degeneration was seen. Dose-dependent effect was only proved for hepatocytes vacuolar degeneration and pycnotic nuclei, while the incidence of hepatocytes edema was higher in the group exposed to a lower dose of the drug. Co-administration of GT decreased the incidence of hepatic abnormalities among the animals exposed to DOX.

**Table 3:** Histopathological hepatic changes in animals exposed to doxorubicin (DOX) with or without green tea (GT)

	n	Eosinophilic degeneration	Parenchymatous degeneration	Vacuolar degeneration	Cellular edema	Pycnotic nuclei of hepatocytes	Necrosis	Inflammatory infiltration
Control	8	0	2	0	0	0	0/0^a^	0/1^b^
1DOX	6	5	5	0	4	0	0/0	0/0
1DOX+GT	6	4	2	1	0	2	0/0	0/1
2DOX	6	5	6	3	2	3	0/0	0/1
2DOX+GT	6	4	4	2	2	0	0/0	0/1

A single animal may be represented more than once in the listing of individual histological changes.

a Massive necrosis/changes limited to single hepatocytes.

b Massive inflammatory infiltration/disseminate mononuclear cells between hepatocytes.

**Fig. 2:**
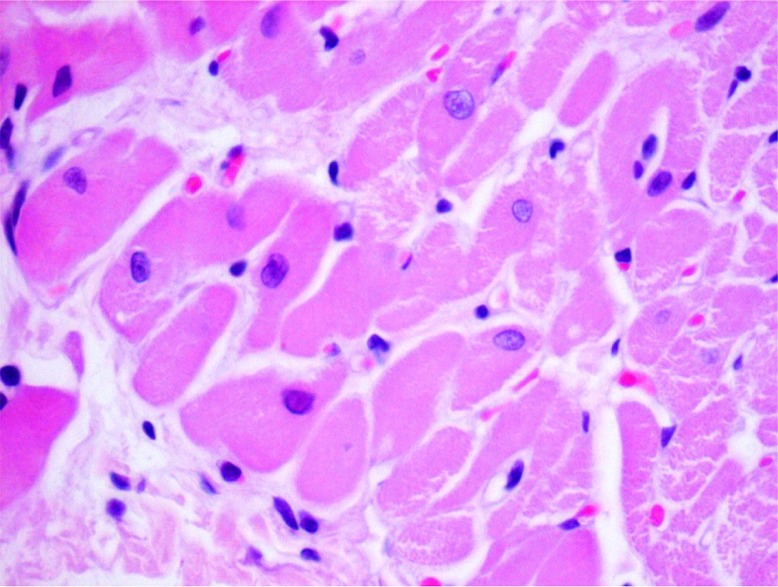
Eosinophilic degeneration, lack of stration, and pycnotic nuclei of cardiomyocytes as well as single intracellular vacuoles (2DOX+GT, H+E, objective mag.×40).

**Fig. 3:**
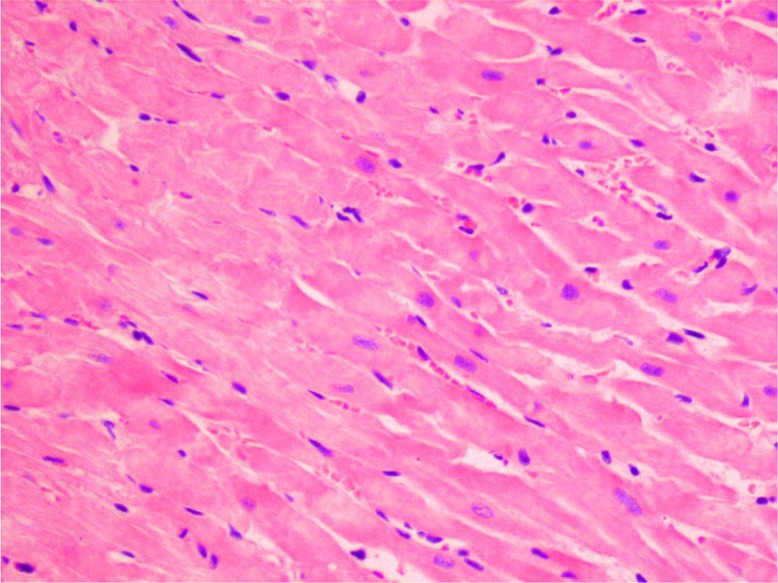
Eosinophilic degeneration of wavy-directed cardiomyocytes with lack of stration (1DOX+GT, H+E, objective mag.×20).

The levels of rat plasma–specific marker for cardiomyocyte necrosis (FABP) and specific marker of heart muscle contractility dysfunction (BNP) were significantly elevated in group 2DOX (Table 4). GT in 2DOX+GT group significantly reduced (vs DOX group) the level of FABP to the normal value and extremely diminished the level of BNP – approximately one order of magnitude below control. Thus, DOX-dependent cardiac necrosis and its GT protection stated in histological examination were confirmed by biochemical marker – FABP (Table 2). Similarly, in these groups, DOX-induced inflammation, eosinophilic degeneration, and interstitial edema were markedly reduced by GT (Table 2, Figs. 2 and 3). Less sensitive and specific serum markers of cardiac necrosis – AST, ALT, CK, and LDH activity – were not elevated (Table 4). Moreover, the activities of these enzymes in groups 2DOX and 2DOX+GT were below control and in most cases these decreases were statistically significant. A significant decrease of ALP activity was found in all xenobiotic-exposed groups. However, no significant changes in the activity of all the aforementioned enzymes were noticed between DOX+GT and DOX groups.

**Table 4:** Serum and plasma markers of heart and liver damage (M±SD)

	FABP (µg/l)	BNP (µg/l)	AST (IU/l)	ALT (IU/l)	CK (IU/l)	LDH (IU/l)	ALP (IU/l)
Control	5.0±1.59	0.40±0.24	104.3±9.07	57.63±7.11	1536.75±343.61	1275.9±596.29	186.25±31.62
1DOX	7.0±1.97	0.54±0.44	96.0±9.03	68.20±13.65	1099.00±421.51	647.8±179.96	89.80±28.86*
1DOX+GT	4.6±1.65	0.37±0.43	99.8±20.28	67.40±7.37*	1271.60±218.570	832.2±164.32	139.00±61.69
2DOX	11.1±3.26*	1.32±0.76*	91.4±45.88	48.80±27.69	953.40±142.70*	519.2±151.58*	84.20±20.09*
2DOX+GT	5.5±2.39^a^	0.05±0.03* a	82.9±14.04*	39.57±20.93	799.43±378.32*	603.6±168.46*	51.71±17.78*

* *p*≤0.05 vs control.

a *p*≤0.05 vs 2DOX.

**Fig. 4:**
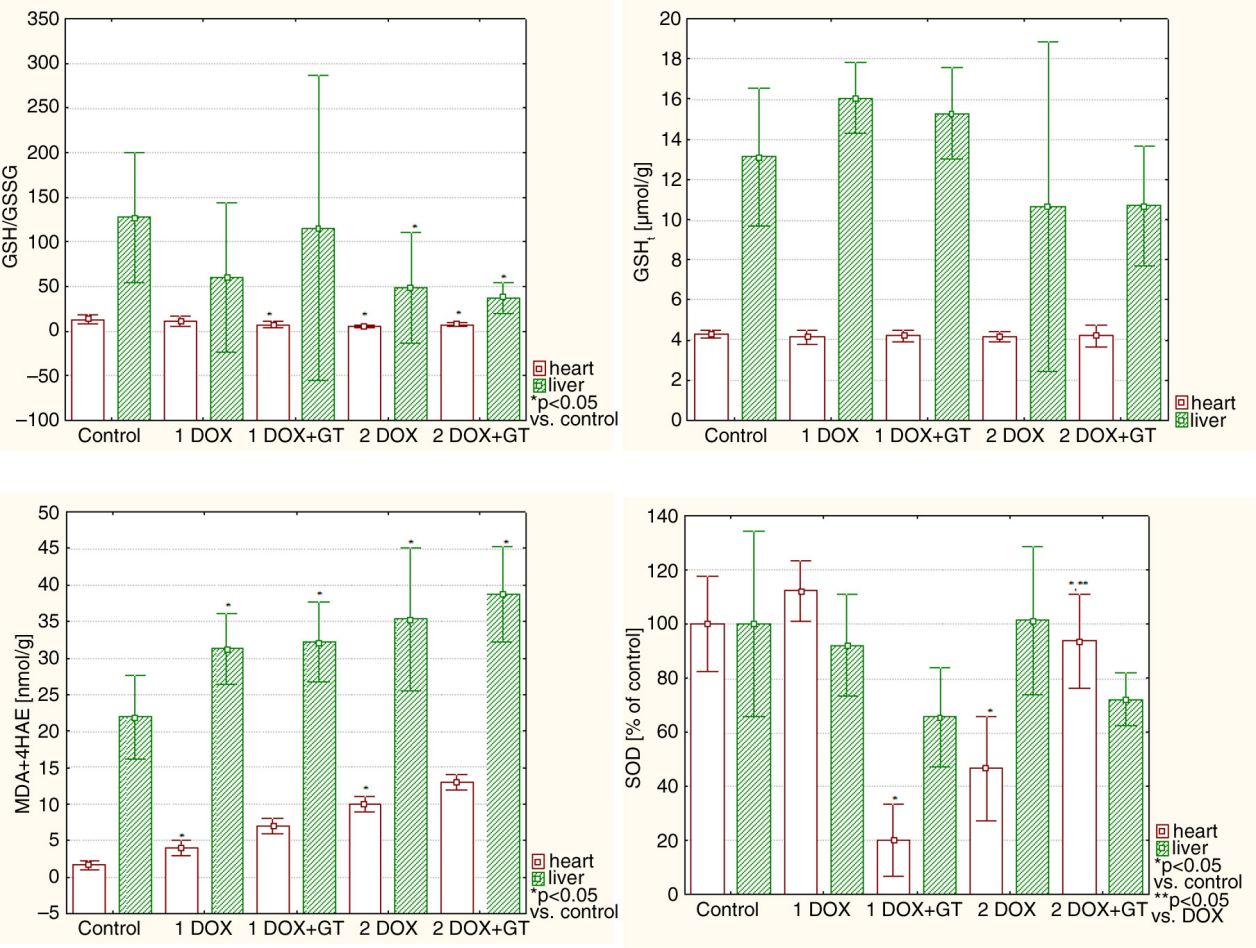
The comparison of relative values of redox equilibrium markers in the heart and liver.

Specific markers for oxidative stress – lipid peroxidation (LPO) products, which are MDA and 4 hydroxynonenal (4HAE) – were significantly elevated in the heart in both groups administered with DOX (1DOX and 2DOX) (Table 5, Fig. 4). DOX-induced lipid peroxidation in such groups was mitigated by GT to the level that is not significantly different compared with the control. The value of cardiac GSH/GSSG ratio was below control in all tested groups, but a statistically significant difference versus control was observed in groups 1DOX+GT, 2DOX, and 2DOX+GT. No differences in cardiac total glutathione (GSH_t_) were noticed. Interesting changes were observed for SOD activity in the heart. A lower dose of DOX insignificantly elevated the SOD activity, but a higher dose of this drug reduces the SOD activity below half the value of the control. GT extremely and significantly reduced the SOD activity in rats receiving a lower dose of DOX, and it was reversed for a higher dose of the drug. DOX-related reduction of SOD activity (group of 2DOX) was significantly elevated toward the normal value when GT was co-administered with DOX.

**Table 5:** Markers of oxidative stress in the heart (M±SD)

	MDA+4HAE (nmol/g)	GSH/GSSG	GSH_t_ (µmol/g)	SOD (% of control)
Control	18.38±5.16	13.33±5.13	4.30±0.20	100.00±17.52
1DOX	28.46±2.41*	11.14±5.14	4.16±0.34	112.22±11.12
1DOX+GT	22.77±3.87	7.25±3.24*	4.22±0.30	19.91±13.29* a
2DOX	24.67±2.25*	5.65±1.61*	4.17±0.26	46.50±19.06*
2DOX+GT	23.00±2.95	7.54±2.53*	4.22±0.53	93.72±17.44^b^

* *p*<0.05 vs control,

a *p*≤0.05 vs 1DOX;

b *p*≤0.05 vs 2DOX.

A statistically significant increase in hepatic MDA+4HAE level was observed in all tested groups compared with the control (Table 6). However, no statistical differences between DOX and DOX+GT were noticed. The GSH/GSSG ratio was significantly reduced in groups of 2DOX and 2DOX+GT, but in this case the difference between 2DOX and 2DOX+GT groups was not statistically significant. No statistically significant differences versus control and DOX groups were observed for hepatic total glutathione and SOD activity.

**Table 6:** Markers of oxidative stress in the liver (M±SD)

	MDA+4HAE (nmol/g)	GSH/GSSG	GSH_t_ (µmol/g)	SOD (% of control)
Control	21.90±5.70	127.36±72.34	13.13±3.34	100.00±34.43
1DOX	31.25±4.91*	59.89±83.48	16.05±1.76	92.18±18.58
1DOX+GT	32.14±5.47*	115.66±171.11	15.29±2.26	65.61±18.24
2DOX	35.32±9.75*	48.59±61.82*	10.65±8.22	101.26±27.27
2DOX+GT	38.78±6.55*	37.72±17.28*	10.70±2.99	72.17±9.65

* *p*<0.05 vs control.

An elevated level of triglycerides was observed (Table 7) in all tested groups compared with the control (1.53 mmol/L). The highest level of triglycerides was noticed in the 2DOX (4.93 mmol/L) and 2DOX+GT groups (9.19 mmol/L). A significant rise of total cholesterol level was revealed in groups 2DOX and 2DOX+GT. In all tested groups, the glucose level was diminished. DOX in both tested doses had no effect on serum uric acid concentration. However, rats co-treated with DOX and GT statistically increased the uric acid level above the control value. Reversely, for urea, DOX elevated the level of this parameter but DOX co-administered with GT quenched this effect. The level of creatinine was slightly reduced but statistically significant when a lower dose of DOX was co-administered with GT.

**Table 7:** Serum markers of metabolism (M±SD)

	Triglycerides (mmol/L)	Total cholesterol (mmol/L)	Glucose (mmol/L)	Uric acid (mmol/L)	Urea (mmol/L)	Creatinine (µmol/L)
Control	1.53±0.31	1.93±0.26	11.73±1.13	0.11±0.02	6.05±0.51	48.62±11.49
1DOX	3.12±1.74*	2.62±0.98	7.66±0.72*	0.15±0.03	5.03±0.84*	31.82±19.45
1DOX+GT	3.13±1.46*	3.40±1.76	9.41±1.03*	0.22±0.09*	5.77±0.83	32.71±5.30*
2DOX	4.93±3.04*	6.70±3.46*	6.65±1.18*	0.18±0.06	9.32±2.39*	52.16±17.68
2DOX+GT	9.19±4.99*	7.36±2.02*	9.07±2.89*	0.51±0.55*	7.24±3.75	45.97±15.91

* *p*<0.05 vs control.

## Discussion

According to our expectations, DOX in the two tested doses induced oxidative damages of lipids in the rat heart and liver. This oxidative stress was inhibited by GT in the heart and was unchanged in the liver. The most important finding of our study is that DOX-induced general toxicity, measured as weekly body mass gain changes, were ameliorated by GT in rats receiving higher dose of DOX (2 mg/k b.w.), but body mass gain was exacerbated by GT in rats administered with a lower DOX dose (1 mg/kg. b.w.). A similar scheme of GT action was observed referring to the heart and liver morphology. GT in these organs ameliorated morphological changes induced by a higher dose of DOX and augmented them in rats treated with a lower dose of the drug. In addition, divergent directions of changes of numerous tested parameters after GT treatment, depending on a given dose of DOX, were found.

The most intriguing puzzle in DOX-related cardiotoxicity is the question as to why cardiomyopathy manifests after months or even years after therapy termination and why its development is clinically silent. It sounds credible that DOX causes permanent oxidative damage of mtDNA, which in turn is responsible for mitochondrial dysfunction (16, 17, 49, 50) and subsequent secondary oversynthesis of ROS (51). This results in a positive feedback loop between mitochondrial DNA damage and mitochondrial ROS overproduction, resulting in cycle augmentation of cardiac failure with the passage of a long time since DOX excretion from the organism (52–54).

For that reason, we assessed the tested parameters 96 h after the final (seventh) dose of the drug, since the intramyocardial half-life of DOX appears to be only 20–30 h (55, 56). In the study, the rats were treated with a cumulative dose of DOX, seven times (1.0 or 2.0 mg/kg), every week, to approach the schedule of the clinical conditions (17). No observed general toxic effect was at 1 mg/kg and general toxic effect without mortality was found at 2 mg/kg (49, 57). However, a similar schedule using even 0.8 mg DOX/kg results in adverse cardiac effects at the histological, ultrastructural, and biochemical levels (15). There is a lot of experimental evidence that ROS triggers many pathological changes in tissues. A high level of ROS may lead to necrosis (58), while a lower one to apoptosis (59, 60). Moreover, persistent, long-term generation of a lower amount of ROS results in organ remodeling (52) because of oversynthesis of collagen (61).

### Effect of DOX

In the heart, hallmarks of histopathological changes, including myocardial necrosis usually with concomitant inflammatory infiltration were found. The cardiomyocytes necrosis was confirmed by an increased blood FABP level in the rats treated with a higher dose of DOX, which was accompanied by an increase in oxidative lipid damages.

**Fig. 5:**
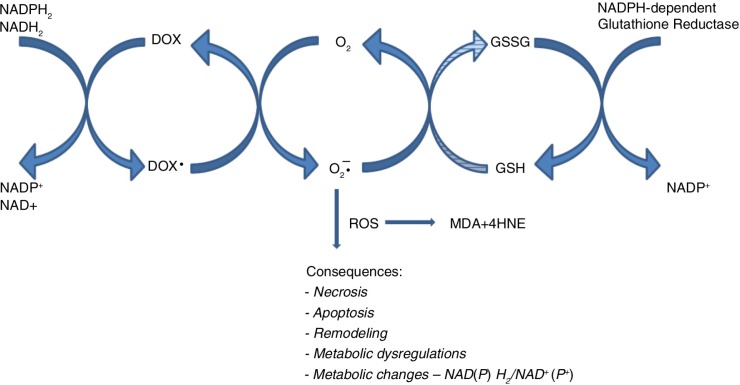
Scheme of postulated doxorubicin-associated NADPH depletion and consequences of ROS overproduction.

In the liver, oxidative stress was relatively higher than in the heart. Subsequent studies can explain if it is related to the higher hepatic activity of NAD(P)H-dependent enzymes, responsible for univalent reduction of DOX and secondary ROS generation (28, 29, 62). This ROS overproduction may cause oxidative stress and may be accompanied by a decrease in the concentration of NADH and NADPH (Fig. 5). Therefore, a univalent reduction of DOX, besides the aforementioned pathological changes, may have significantly affected the metabolic changes. Free radicals (O2*−) inhibit aconitase, and MDA and 4HNE – lipid peroxidation products – inhibit other enzymes of the Krebs cycle. On the contrary, NADH and NADPH are involved in anabolism and catabolism reactions and may be implicated in glucose and lipid metabolism. Unexpectedly, no necrosis, apoptosis and collagen oversynthesis were histologically detected in the liver of all groups when DOX was given. Moreover, in the liver of all drug-exposed animals, there were no significant changes, in glycogen, collagen, or lipid distribution, compared with the control. However, significant unfavorable changes were observed in blood glucose and triglycerides. A decrease in glucose and an increase in triglyceride level were found in groups receiving DOX in both doses. These changes were accompanied by a loss of body weight, which was statistically significant in rats administered with a higher dose of DOX.

The observed reduction of body weight gain (1DOX) and even a decrease in group 2DOX is probably secondary to the reduction of the fat tissue. The biochemical signal for decay of the fat tissue is probably caused by a lower level of glucose, which was found in the blood of these rats. It is consistent with the study of Berthiaume and Wallace (63), which reveals a decrease in transcript associated with fatty acid metabolism and an increase in the expression of several glycolytic genes, suggesting a switch in substrate utilization of the rat heart 5 weeks after termination of the treatment with a cumulative dose of DOX – 12 mg/kg b.w. It is in line with Mandziuk et al. (50), who revealed an increase in cell glucose transporter after DOX treatment. It can be also postulated that an increase in blood triglycerides level in the group receiving DOX may be secondary to the inhibition of mitochondrial complexes of electron transport by this drug (17). The inhibition of mitochondrial electron transport chain leading to an increase in NADH may result in a negative feedback with ß-oxidation. Next, inhibition of ß-oxidation should cause steatosis of the liver. However, steatosis was not observed in the studied rats, which is consistent with our previous study (cumulative dose 10.8 DOX kg/b.w.), where significant hepatic triglycerides were reduced (64, 65).

### The effect of GT on DOX-induced changes

In the current study, a caffeine-free standardized extract of GT leaves was freely taken by rats with drinking water in an approximately daily dose of 16.3 mg/kg b.w. It is a relatively safe dose. In randomized studies with healthy volunteers, the tested doses of catechins (such as EGCG) 1.7–6.8 mg/kg administered every day for 4 weeks were safe and well tolerated (66). And NOAEL for obese dogs was estimated as 500 mg of catechins (80% EGCG) per kg/day (34).

DOX-induced lipid peroxidation in the rat heart and liver was protected by GT in the heart and was unchanged in the liver. Similar data were presented in a study by Zheng et al. (67), who found protection of GT against DOX-induced cardiomyocytes injury. It is very interesting to note that GT protects from DOX-induced lipid peroxidation in cardiomyocytes but at the same time it reduces the level of GSH/GSSG ratio and leads to a collapse of SOD activity (1DOX+GT) or normalized SOD activity in group 2DOX+GT compared with the 2DOX group. The basis of these changes is unclear; however, it seems that GT in the adaptive mechanism activates GSH utilization as an antioxidant agent in the protection from oxidative stress. Protective properties of GT found in rats administered with a higher DOX dose are in line with the results obtained by Li et al. (42) and Khan et al. (43). However, in those studies, a single dose of DOX, close to the LD50 value, was used, or they were conducted in cellular cultures. The question arises as to why GT protects from DOX-induced heart lipid peroxidation with no protection against the liver lipid peroxidation. In light of the obtained results, it is difficult to answer the above question and further studies are necessary.

There were similar effects of GT on the heart and liver morphology in rats treated with both doses of DOX. In both organs, GT has a beneficial effect on the histological changes in rats co-treated with a higher dose of DOX while it slightly exacerbated the histopathological abnormalities induced by a lower DOX dose. In the heart, fresh necrosis induced by DOX (2 mg/kg. b.w.) was reduced by GT. This protective effect was confirmed by the assessment of FABP (2DOX+GT vs 2DOX). In the study by Sayed-Mahmed et al. (68), inhibition of gene expression of heart FABP in DOX cardiomyopathy rat model was found. Consequently, it should be manifested in a lower level of blood FABP in contrast to our study, which probably results from a schedule for different doses. The level of other unspecific cell necrosis markers assessed in these groups (AST, ALT, CK, and LDH) was reduced. Such a reduction is not important in clinical practice but it was sometimes observed during exposition to some poisons. GT extract significantly protects from DOX-induced disturbance in contractility (2DOX+GT vs 2DOX), which was stated in the blood BNP changes.

There were different effects of GT on general toxicity expressed as gained body weight in rats treated with two doses of DOX. In rats receiving lover dose of doxorubicin a slightly negative effect of green tea was observed but green tea reveal beneficial effect in rats administered with higher dose of doxorubicin. Cholesterol elevation was also revealed in rats treated with a higher dose of DOX. GT has an insignificant impact on glucose cholesterol and triglycerides level, although GT can modulate the activity of enzymes involved in lipids synthesis, for instance HMG-CoA reductase (40, 69).

In conclusion, according to our expectations, DOX in both doses caused oxidative stress in the heart and higher stress in the liver, which was inhibited by GT in the heart and was unchanged in the liver. DOX-induced adverse changes in body mass and histopathological changes in the heart and in the liver were mitigated by GT at a higher dose of DOX and augmented in rats treated with a lower dose of the drug. There were multidirectional effects of GT on blood metabolic parameters changed by DOX. Among all tested biochemical parameters, significant protection of GT against DOX-induced changes was revealed in the case of blood FABP and BNP and cardiac SOD level. The results reveal a divergent effect of GT dependent on DOX cumulative dose. Thus, subsequent studies are necessary to assess the effect of GT on DOX-dose dependent disturbance in cardiac function in long-term periods since termination of DOX injections. The main limitation of the study is the low number of evaluated animals and species differences between humans and rats. For such a reason, the obtained result cannot be directly applied in a daily clinical practice.
